# The Impact of the Ukraine Conflict on the Quality of Life of the Young Population in Romania from a Societal Security Perspective

**DOI:** 10.3390/healthcare13020156

**Published:** 2025-01-15

**Authors:** Flavius Cristian Mărcău, Cătălin Peptan, Floris Petru Iliuta, Marian Emanuel Cojoaca, Alina Magdalena Musetescu, Alina Georgiana Holt, Ina Raluca Tomescu, Genu Alexandru Căruntu, Victor Gheorman

**Affiliations:** 1 Faculty of Educational Sciences, Law and Public Administration, “Constantin Brâncuși” University of Târgu Jiu, 210185 Târgu Jiu, Romania; flavius.marcau@e-ucb.ro (F.C.M.); catalin.peptan@e-ucb.ro (C.P.); alina.holt@e-ucb.ro (A.G.H.); ina.tomescu@e-ucb.ro (I.R.T.); 2 Department of Psychiatry and Psychology, Discipline of Psychiatry, Faculty of Dental Medicine, ‘Carol Davila’ University of Medicine and Pharmacy, 010221 Bucharest, Romania; 3 Department of Psychiatry, ‘Prof. Dr Alexandru Obregia’ Clinical Hospital of Psychiatry, 041914 Bucharest, Romania; 4 National Health Insurance House (CNAS), Titu Maiorescu University, 040051 Bucharest, Romania; marian.cojoaca@utm.ro; 5 Victor Babeș Hospital for Infectious and Tropical Diseases, 030303 Bucharest, Romania; alina.musetescu@utm.ro; 6 Faculty of Medicine, Titu Maiorescu University, 040051 Bucharest, Romania; 7 Faculty of Economics, “Constantin Brâncuși” University of Târgu Jiu, 210185 Târgu Jiu, Romania; genu.caruntu@e-ucb.ro; 8 Department of Psychiatry, University of Medicine and Pharmacy of Craiova, 200349 Craiova, Romania; victor.gheorman@umfcv.ro; 9 Department of Psychiatry I, Craiova Clinical Neuropsychiatry Hospital, 200473 Craiova, Romania

**Keywords:** security, quality of life, Ukraine conflict, young population Romania

## Abstract

Background/Objectives: This study examines the perception of young Romanians (aged 18–35) regarding the Ukraine conflict’s impact on Romania’s national security and quality of life. It focuses on societal security dimensions, analyzing the conflict’s regional and international implications, alongside sentiments toward global support for Ukraine. Methods: Data were collected via structured questionnaires administered to 848 participants in 2024 and 747 in 2022. Respondents’ perceptions of the Ukraine conflict, its influence on national security, and the direct consequences for quality of life were evaluated using the WHOQOL-BREF instrument. Statistical analyses (descriptive, bivariate, multivariate) were employed to explore variations across socio-demographic factors. Results: The findings indicate that young Romanians appreciate international solidarity with Ukraine but remain cautious about military escalation. While the conflict heightened perceptions of personal and national insecurity, WHOQOL-BREF assessments revealed significant declines in psychological, social, and environmental quality of life indicators from 2022 to 2024. Variations were observed based on age, gender, education level, and residence, with young rural women and those with lower educational attainment experiencing the most pronounced impacts. Conclusions: The study underscores the dual challenges of maintaining regional stability and addressing the socio-psychological fallout of conflicts. Despite resilience mechanisms tempering subjective perceptions, objective measures indicate deteriorations in the quality of life among Romania’s young population. These findings emphasize the need for targeted policies to support vulnerable groups through mental health initiatives, social support systems, and equitable access to resources.

## 1. Introduction

In a period marked by geopolitical conflicts and international tensions, the war initiated by the Russian Federation in Ukraine has triggered a series of far-reaching consequences. Romania, as a member of the North Atlantic Treaty Organization (NATO) and a neighbor to Ukraine, faces substantial challenges regarding both national security and the well-being (quality of life) of its population.

In this study, we draw on the concept of societal security, a key framework of the Copenhagen School developed by Barry Buzan and Ole Wæver. This perspective extends beyond the strictly military dimension of security, focusing on how societies preserve their identity, values, and cohesion when confronted with internal and external threats. According to Buzan and Wæver, societal security involves safeguarding the elements that give cohesion and meaning to a community—ranging from shared culture and language to the sense of belonging and the stability of social institutions. Consequently, in the context of the conflict in Ukraine, a societal security perspective enables us to explore not only the military and geopolitical dimensions but also how social cohesion, perceptions of safety, and the well-being of young people in Romania are affected.

Our initial study [1], which examined Romanian public perceptions of how the conflict impacts quality of life, revealed an increase in fear, worry, and anxiety. These responses reflect broader global tensions as well as a collective desire for peace and stability.

Over two years after the onset of this crisis, it is essential to reassess the situation in light of shifts in the conflict’s dynamics. The geopolitical context has changed considerably [2], and Romanian society’s adaptation to these uncertainties and tensions indicates a more mature response to regional and international developments. NATO’s expanded role in Eastern Europe, the economic sanctions imposed on the Russian Federation, and worldwide diplomatic efforts have generated a new environment with direct implications for Romanians’ sense of security and overall well-being.

This research is driven by the need to understand how young Romanians (aged 18–35), a vital demographic for the country’s future, perceive and respond to these changes. By focusing on their sense of security and quality of life, the study not only provides insights into the immediate consequences of the conflict but also anticipates potential trends and policy needs in Romania’s domestic and foreign affairs.

Set against an unpredictable global backdrop—including a pandemic that tested societal resilience and heightened geopolitical tensions [3]—the pursuit of relevant, up-to-date research is crucial. Our work continues previous efforts to map the effects of conflicts with escalation potential, while also enhancing our understanding of how societies respond to these challenges.

Through this study, we aim to further investigate how the ongoing conflict in Ukraine has influenced young people in Romania. The primary goal is to analyze the evolving impact of this conflict on their sense of security and quality of life. Considering the developments over the past two years—which have shifted the geopolitical landscape and introduced new challenges for regional security and stability—understanding how young Romanians adapt is increasingly important.

Our motivation stems from the need to evaluate shifting public opinion in the face of major geopolitical events, as well as to gauge the resilience and adaptability of Romanian society. By examining these perceptions, this research seeks to bridge academic inquiry, societal interests, and policymaking, offering insights to help address present and future challenges in a strategic and informed manner.

Within this context, we investigate whether the changes in the geopolitical environment have affected the perceived quality of life among young Romanians. Despite the prolonged conflict in Ukraine generating negative regional repercussions, our hypothesis is that, as of the data collection period (15 March–15 April 2024), there is an objectively significant decline in the quality of life of Romania’s youth compared to the start of the conflict in 2022 [1]. However, subjectively, this decline is not perceived as substantial, due to the development of resilience within this demographic.

### Literature Review

Military conflicts have drawn the attention of researchers from various fields, as their impact goes beyond the geopolitical sphere and extends to the social, economic, and psychological structures of the affected communities. The conflict in Ukraine, triggered by the annexation of Crimea by the Russian Federation in 2014 and intensified through the “special military operation” launched on 24 February 2022, has been a focal point for numerous studies focused on security, geopolitics, and the social consequences of such crises [4,5,6,7]. Findings from these studies have highlighted that such conflicts can produce long-term effects, leading to profound restructurings in the social and economic domains, as well as significant changes in the psychological state of the population [8,9,10]. In regions affected by war, resource shortages, forced relocations, uncertainty, and fear can generate chronic stress, mental health decline, and reduced access to essential services [11,12,13,14].

Quality of life is a multidimensional construct that includes both objective elements (income, housing, environmental conditions) and subjective components (life satisfaction, psychological well-being, sense of security) [15]. In this regard, the World Health Organization (WHO) has developed standardized instruments, such as the World Health Organization Quality of Life-BREF (WHOQOL-BREF), to assess four fundamental dimensions of quality of life: physical health, psychological health, social relationships, and the environment [15]. Studies conducted in conflict zones have demonstrated the utility of WHOQOL-BREF in highlighting how geopolitical tensions can affect various aspects of well-being. For instance, physical health may be influenced by disruptions in access to medical services, while psychological health may suffer due to exposure to trauma, feelings of insecurity, or chronic stress. Social relationships tend to become more fragile, especially when populations are forced to migrate and communities are destabilized. Finally, the environment, as a dimension of quality of life, encompasses factors such as physical safety, resource accessibility, and socio-economic infrastructure, all of which can be severely affected in an area bordering a conflict [13,14,15].

The specialized literature points out that young people represent a group with specific vulnerabilities in the context of conflict [16,17,18]. They are in a transitional stage toward adulthood, which involves identity formation, building educational and career paths, as well as developing social and support networks. Sudden changes, such as political and economic instability or threats to security, can have long-term repercussions on their development [19,20,21,22]. However, the same population segment can also demonstrate remarkable resilience, particularly when they benefit from family and community support, access to accurate information, and adaptation resources (psychological counseling, youth support programs, civic engagement).

Previous studies indicate that coping mechanisms and adaptation strategies can vary depending on factors such as education level, socio-economic status, social support network, and proximity to conflict [16,17,18]. Additionally, access to online platforms and social media plays an increasingly important role in how young people construct their perception of conflict, seek support, or express their fears. This can be both advantageous, by creating virtual support communities, and a source of misinformation and amplified anxieties.

Recent research has shown that the young population in Romania experienced a decline in quality of life even in the early stages of the conflict in Ukraine, indicating an immediate reaction to geopolitical tensions [1,23,24,25]. Among the factors identified in the literature are concerns about Romania’s potential long-term involvement in the conflict, the influx of refugees, and additional pressures on education, healthcare, and labor market systems [26,27,28]. Moreover, the perception of insecurity can be amplified by the media and the online environment, which continuously expose young people to information about the conflict’s evolution and its consequences.

Although the literature provides a broad understanding of the effects of military conflicts on civilian society and adaptation strategies, a gap remains in our knowledge regarding the specific impact of the conflict in Ukraine on young people in neighboring countries such as Romania. Many existing studies focus on populations directly affected by the conflict (for example, those in occupied regions or refugees), without paying close attention to those living in areas adjacent to the conflict, who may still experience significant indirect effects [26,27,28].

To address this research gap, the present study aims to investigate how young people in Romania perceive the effects of the conflict in Ukraine on their quality of life, using the WHOQOL-BREF instrument. This integrated approach responds to the need for continual updates on data and analyses regarding the ever-changing geopolitical consequences [29,30,31]. Identifying changes in the dimensions of quality of life—physical health, psychological health, social relationships, and environment—can provide a comprehensive picture of young people’s well-being and their specific vulnerabilities. Furthermore, understanding risk and protective factors in this population can help develop practical recommendations for decision-makers and policy initiatives.

Given that the relationships among security policies, the geopolitical framework, and quality of life are extremely dynamic, our analyses and conclusions may serve as a starting point for further investigations and the adoption of measures to support young people in a context of regional instability. Through the contribution of this study, we seek to offer valuable data and interpretations for understanding the impact of the conflict in Ukraine on Romanian youth, thus contributing to a richer academic dialogue and the formulation of appropriate interventions in social and political domains.

## 2. Materials and Methods

### 2.1. Participants

Our study is based on the perceptions of individuals surveyed at two different points in time, specifically: a group of 848 participants aged 18 to 35 who completed an online questionnaire distributed via Facebook and various websites between 15 March and 15 April 2024; and a group of 747 participants who completed the questionnaire in 2022, starting from the fifth day after the onset of the conflict in Ukraine, during the period of 1–17 March 2022.

The samples were divided into two age categories (18–25 and 26–35 years), reflecting diverse opinions within this demographic group. The selection of this age segment was aligned with the vision of the Ministry of National Defense regarding the enhancement of Romania’s defense capabilities [32] and with national legislation on preparing the population for defense [33]. The samples were split into the two categories, centered around the age of 26 (considered a benchmark for assuming full responsibility for supporting young individuals), in accordance with Article 499, Paragraph (3), of the Romanian Civil Code [34].

The selection of participants adhered to strict ethical principles, ensuring complete anonymity and confidentiality of the collected data. The questionnaire did not include any information that could identify respondents, underscoring the commitment to protecting their identity. Participation was voluntary, unpaid, and anonymous, with informed consent obtained before completing the questionnaire. These measures created a safe environment for participants, enabling the collection of relevant data to analyze the impact of the conflict in Ukraine on the perceptions and attitudes of young people, providing valuable insights two years after the outbreak of the war.

### 2.2. Procedure

The data were collected using a questionnaire created on the Google Forms platform, chosen for its flexibility and accessibility for both researchers and respondents. The structure of the questionnaire was designed to be intuitive, aiming to encourage a high number of participants from the target age group (18–35 years). In conducting this wave of research (2024), we sought, as much as possible, to maintain a methodology similar to that of the previous study in 2022, to ensure the comparability of results between the two periods.

To ensure a broad and relatively comparable distribution with that of 2022, the link to the questionnaire was shared on various online platforms, including social networks and thematic groups, aiming to attract participants with diverse demographic and geographical profiles. Aware that online distribution can lead to a certain degree of self-selection (e.g., individuals more active on social media or interested in the topic are more likely to respond), we attempted to distribute the link in communities similar to those targeted in 2022, thus reducing the risk of significant differences in the sample composition. Additionally, the redistribution of the link by third parties was allowed, which increased dissemination but also resulted in reduced control over the directions of spread.

To address potential biases associated with this recruitment method, we monitored response rates and the demographic profile (age, gender, region) of participants throughout the data collection period. This monitoring allowed us to identify any imbalances and adjust the distribution strategy (e.g., by promoting it in underrepresented regions), maintaining a structure as similar as possible to that of 2022.

A crucial step in the procedure was the initial screening of participants through an eligibility statement placed at the beginning of the questionnaire: “I am at least 18 years old and no older than 35, and after reading the informed consent, I agree to participate in this study”. This step was mandatory to ensure that all respondents met the age criteria and acknowledged the informed consent before proceeding with the questions. In this way, participation was based on voluntary and informed consent, adhering to ethical and confidentiality standards.

Through these measures, we aimed to maintain a high level of methodological consistency between the two waves of research (2022 and 2024), both in terms of distribution methods and sample structure. This approach allowed us to rigorously compare the data and more clearly highlight any potential developments or changes in the perceptions of young people in Romania.

### 2.3. Measurements

The questionnaire was structured into distinct sections, with a total of 26 questions from the WHOQOL-BREF instrument, developed by the World Health Organization, which is internationally recognized for assessing quality of life. This instrument analyzes four major domains: the Physical Domain, which focuses on aspects of physical well-being, such as pain, energy, sleep quality, and mobility; the Psychological Domain, which examines emotional and mental well-being, including feelings, learning ability, memory, concentration, self-esteem, and self-image; the Social Domain, which evaluates the quality of interpersonal relationships and social support received; and the Environmental Domain, which analyzes living conditions, such as safety, home comfort, financial situation, access to health services, and recreational options.

Each question in the WHOQOL-BREF is rated on a scale from 1 to 5, where a higher score indicates a better quality of life. To calculate the score for each domain, the average of the corresponding responses is taken and multiplied by 4, transforming the score to a scale from 4 to 20, allowing for comparison with other similar measures, such as WHOQOL-100. This structure allowed for a detailed assessment of the conflict’s impact on participants’ quality of life [35].

### 2.4. Statistical Data Analysis

For the data analysis obtained from the administered questionnaire, Microsoft Office Professional Plus 2021 and IBM SPSS Statistics 26 were used, with data organized in an Excel file for easier visualization, extraction, and detailed statistical analysis.

Bivariate analysis was conducted to explore relationships between key variables (such as physical and psychological health, quality of social relationships, environmental health, quality of life, and health satisfaction) and socio-demographic factors (age, gender, residence, education). This helped highlight how respondents’ perceptions varied based on these factors.

## 3. Results

The study was conducted based on a questionnaire that was administered to 848 individuals for 2024, and 747 individuals for 2024, whose socio-demographic data are presented in Table 1.

### 3.1. Subjective Quality of Life Assessment

The analysis of the survey results reveals significant differences between various socio-demographic groups regarding their perceptions of the conflict in Ukraine and its implications for Romania. These differences are highlighted in Table 2.

The analysis of perceptions regarding the influence of the conflict in Ukraine indicates different trends across socio-demographic groups. Among the 18–25 cohort, a small change was noted from 2022 to 2024; however, statistical tests (*p* > 0.05) show that this difference was not significant. Similarly, for the 26–35 age group, no statistically significant changes were identified (*p* > 0.05).

In contrast, gender differences were more prominent in 2024: men reported a significantly higher perceived impact of the conflict than women (t = 1.96, *p* = 0.05 for men; t = −4.14, *p* < 0.01 for women). This result suggests a consistent pattern in which men feel the effects of the conflict more intensely.

Residential environment also influences perceptions: in 2024, rural residents reported a significantly higher perceived impact (t = −2.12, *p* = 0.03), indicating a greater vulnerability among this population. Regarding education level, although minor fluctuations were observed, these differences were not statistically significant (*p* > 0.05). Hence, individuals with university education appeared to maintain a relatively stable perception of the conflict’s influence, potentially reflecting a less reactive stance toward external events stemming from the crisis in Ukraine.

Finally, more complex analyses (e.g., multiple regression) highlight interesting interactions between variables: the interaction of age and education was statistically significant in both 2022 and 2024 (coef. = 0.394, *p* < 0.05 for 2022; coef. = 0.432, *p* < 0.05 for 2024), indicating a joint effect on how the conflict is perceived. Gender differences remained particularly noticeable in logistic models, where male respondents were associated with a stronger perception of the conflict’s influence (coef. = −0.516, *p* < 0.001).

### 3.2. Objective Assessment of Quality of Life

#### 3.2.1. General Results

Table 3 presents descriptive statistics for the four dimensions of well-being: physical (PHYS), psychological (PSYCH), social (SOCIAL), and environmental (ENVIR), measuring perceived levels of well-being among a sample of 848 respondents in 2024 and 1193 respondents in 2022.

The descriptive statistics in Table 3 offer a comparative overview of various health dimensions and quality of life between 2022 and 2024. For the PHYS dimension, a noticeable improvement was evident in 2024, with a mean score of 66.76 compared to 61.64 in 2022. This increase reflects a broader trend toward enhanced physical health among respondents over the period. The standard deviation also rose slightly in 2024, indicating a more significant variability in physical health perceptions.

In the PSYCH dimension, the mean scores remain relatively stable, with a slight decline from 73.16 in 2022 to 71.37 in 2024, suggesting a minor decrease in psychological well-being over the observed period. The standard deviation increased from 15.05 in 2022 to 21.33 in 2024, which implies a broader range of psychological health perceptions among respondents in the latter year.

The social dimension scores demonstrate a reduction in mean values from 74.62 in 2022 to 68.64 in 2024, reflecting a decrease in social well-being or connectedness. However, the standard deviation has remained almost constant, suggesting that the variability in social well-being perceptions across respondents has not significantly changed.

For the ENVIR, mean values similarly declined slightly from 71.30 in 2022 to 68.09 in 2024, indicating a minor drop in environmental quality or satisfaction. The standard deviation shows a marginal increase, pointing to a slight growth in the dispersion of environmental well-being scores.

#### 3.2.2. Bivariate Analysis

Table 4 details the results of the bivariate analysis, highlighting variations in the perceptions of physical and psychological health, social relationships, environmental health, quality of life, and health satisfaction based on demographic and social variables such as age, gender, living environment, education level, professional status, and income level for the years 2022 and 2024.

The previous table presents a comparative analysis between 2022 and 2024 based on WHOQOL-BREF scores and subjective responses to questions about quality of life and satisfaction with health. Significant changes in young people’s perceptions of their quality of life, influenced by the conflict in Ukraine, are highlighted.

In the physical health dimension, objective data show an overall decline in scores in 2024 compared to 2022 across all demographic categories. For instance, the 18–25 age group recorded a significant drop in the average score, from 71.20 in 2022 to 63.74 in 2024, indicating a reduction in physical well-being. For the 26–35 age group, the decline was less pronounced, from 71.32 in 2022 to 69.91 in 2024. Gender differences are evident; men maintained relatively stable scores, with a slight increase from 75.20 in 2022 to 75.77 in 2024, while women reported a larger decline, from 69.66 in 2022 to 64.41 in 2024. A more significant drop was observed in rural areas (65.39 in 2024 compared to 71.89 in 2022) than in urban areas (67.34 in 2024 compared to 70.82 in 2022), suggesting a higher vulnerability among rural residents. Similarly, respondents with university education experienced a sharper decrease (50.80 in 2024 compared to 70.81 in 2022) than those with middle and high school education (64.01 in 2024 compared to 71.76 in 2022), indicating greater concern in the former group.

The psychological health dimension showed a similar trend. Average scores dropped significantly for the 18–25 age group, from 76.54 in 2022 to 67.61 in 2024, reflecting a deterioration in mental health. For the 26–35 age group, scores remained relatively stable (79.10 in 2022 to 75.29 in 2024). Gender differences indicate declines for both men (73.97 in 2024 compared to 80.45 in 2022) and women (76.33 in 2022 to 69.85 in 2024). Rural areas reported a significant decrease in scores (80.37 in 2022 compared to 70.86 in 2024), with an even more pronounced decline observed in urban areas. Important distinctions were also noted in education levels, with those having middle and high school education showing a sharper decline (68.27 in 2024 compared to 77.50 in 2022).

Social relationships were significantly affected. Average scores dropped for all categories, especially for young people aged 18–25 (73.55 in 2022 to 66.07 in 2024). The 26–35 age group saw a less severe decline (76.34 in 2022 to 71.32 in 2024). Gender differences are notable, with men experiencing a smaller drop (75.50 in 2022 to 70.76 in 2024) compared to women (74.26 in 2022 to 67.39 in 2024). Rural residents reported a more significant decline (74.51 in 2022 to 69.31 in 2024) than urban residents (74.77 in 2022 to 67.68 in 2024), indicating additional vulnerabilities in rural areas. Those with middle and high school education also reported a sharper decline (74.20 in 2022 to 67.03 in 2024) than those with higher education (74.97 in 2022 to 70.68 in 2024).

In the environment dimension, scores showed generalized declines between 2022 and 2024. Young people aged 18–25 experienced a drop from 72.32 in 2022 to 67.31 in 2024, while the 26–35 age group saw a less severe decrease (69.66 in 2022 to 68.90 in 2024). Gender differences were evident, with men reporting stable scores (73.49 in 2022 to 73.18 in 2024), while women experienced a larger decline. Rural areas reported a more pronounced deterioration in perceptions of environmental quality (71.54 in 2022 to 67.57 in 2024) compared to urban areas (71.15 in 2022 to 68.46 in 2024). Respondents with middle and high school education experienced a steeper decline (72.52 in 2022 to 67.25 in 2024) compared to those with higher education (70.26 in 2022 to 69.16 in 2024).

Subjective responses to questions about quality of life and satisfaction with health indicated relatively stable perceptions over time. For example, among the 18–25 age group, quality of life was rated at 3.41 in 2024 compared to 3.29 in 2022. The 26–35 age group showed nearly constant scores. Differences in gender and residence were less evident in subjective responses, suggesting a discrepancy between subjective perceptions and objective scores.

Overall, compared to 2022, the data highlight a generalized deterioration in the objective dimensions of quality of life, especially in physical health, psychological health, and social relationships. This trend is more pronounced among young people in rural areas, women, the 18–25 age group, and those with middle and high school education.

### 3.3. Analysis of Sampling Error Across Survey Questions

In this study, we calculated the sampling errors for each question, for the data collected both in 2022 and 2024, to provide additional context for interpreting the results and to strengthen methodological rigor.

For 2022, the overall sampling error, calculated at a 95% confidence level, was ±3.59%, while for 2024, it was ±3.37%. The difference between these values is due to the increased sample size in 2024, which contributes to reducing the uncertainty associated with the estimates.

For each question, the sampling error was determined based on the observed response proportions. These errors provide a confidence interval within which the actual population values are estimated to lie. Thus, interpreting differences between the two periods must be performed with caution, considering these error margins.

## 4. Discussion

Analyzing the impact of the conflict in Ukraine on the population of Romania is essential for understanding the psychosocial dimension of modern warfare and its effects on civilian populations [26,36,37,38]. In a context where the front lines are not limited to the battlefield but also extend into informational and psychological spaces, the study addresses a component often neglected in traditional analyses of armed conflicts, namely the well-being and safety of the population directly or indirectly affected [39,40,41].

As a NATO member and neighbor to Ukraine, Romania finds itself in a unique position, needing to balance the obligations of the alliance with the direct challenges posed by geographical proximity to the conflict zone [42,43]. This status generates a complex mix of fears, uncertainties, and expectations among the population, with a direct impact on the perception of quality of life and the sense of security [40,44,45,46].

One of the immediate effects of the conflict on the population of Romania is an increase in the level of anxiety and a sense of insecurity [1]. Fears regarding the possibility of the conflict’s expansion and direct impact on Romania [37], as well as concerns about the economic and social consequences of the conflict, have led to a general state of unrest. This is not limited to potential military risks but also includes fears related to refugee flows, energy security, and economic stability [47].

### 4.1. Synthesis of Results and Comparison with the Specialized Literature

The hypothesis of this research posits that, at the time of the study (15 March–15 April 2024 *), the quality of life of the young population in Romania experienced a significant objective decline compared to the onset of the conflict in Ukraine in 2022 (**) [1]. However, this decline is not subjectively perceived as significant due to the development of resilience mechanisms among the population. While the overall trend supports this hypothesis, a detailed analysis of the data (Table 3 and Table 4) reveals variations across the dimensions of quality of life (QoL) and socio-demographic categories, influenced by external factors such as the Ukraine conflict.

#### 4.1.1. Physical Health Dimension

Descriptive statistics in Table 3 indicate a general improvement in the physical health dimension, with the average score rising from 61.64 in 2022 to 66.76 in 2024. This trend is supported by research showing an overall increase in health-related quality of life during recovery from stressful events [48,49,50].

This positive trend suggests resilience among respondents, although a larger standard deviation in 2024 reflects greater variability in perceptions of physical health. These results align with the literature highlighting the influence of psychological and social factors on physical health perception [51,52,53].

However, Table 4 reveals notable disparities: young people aged 18–25 experienced a sharp decline (71.20 ** to 63.74 *), in contrast to stable or slightly improved scores among men (75.20 ** to 75.77 *). Gender and age disparities in physical health are frequently reported in studies exploring the impact of socio-political stressors [54,55].

Women (69.66 ** to 64.41 *) and rural populations (71.89 ** to 65.93 *) were more affected, consistent with the literature emphasizing unequal resilience in physical health among populations impacted by conflicts. These observations are supported by studies highlighting health inequalities based on gender and residence [56,57,58].

#### 4.1.2. Psychological Health Dimension

The psychological health dimension showed minor declines, with average scores decreasing from 73.16 in 2022 to 71.37 in 2024 (Table 3). The increased standard deviation (15.05 ** to 21.33 *) signals greater disparities in psychological well-being. These results are supported by studies indicating that chronic stress can lead to more pronounced individual differences, exacerbating mental health disparities [59,60,61,62].

Table 4 underscores significant challenges for young people aged 18–25, whose scores dropped drastically (76.54 ** to 67.61 *), and for women (76.33 ** to 69.85 *), compared to less severe declines among men and urban populations. Research shows that vulnerable groups, such as young people and women, are more exposed to mental health risks under prolonged stress and conflict, with higher prevalence rates of depression and anxiety symptoms [63,64,65]. Furthermore, social and economic vulnerabilities intensify the negative impact on women’s and young people’s mental health [66,67].

These findings are consistent with research highlighting the impact of prolonged stress and conflict on mental health, particularly among vulnerable groups. Daily stressors in conflict contexts are known to exacerbate psychological disorders, including anxiety and depression, especially in individuals directly exposed to war [64,68]. Prolonged exposure to stress can amplify pre-existing symptoms and increase susceptibility to new mental health disorders [69,70,71].

#### 4.1.3. Social Dimension

The social dimension experienced the largest overall decline, with average scores dropping from 74.62 in 2022 to 68.64 in 2024 (Table 3). Variability remained constant, indicating evenly distributed declines across the population. Studies suggest that economic and social crises contribute to the deterioration of social well-being, particularly in perceived quality of life and social support [72,73].

Table 4 highlights that young people aged 18–25 were the most affected (73.55 ** to 66.07 *), and women and rural residents also reported significant declines. The lack of strong support networks can exacerbate vulnerability in these groups, especially under prolonged stress conditions [74,75,76].

The stability observed among men and urban respondents reflects the mitigating role of better access to support networks in urban areas, a finding supported by global research on social relationships during crises. Studies show that social support has a protective effect against stress by mitigating its impact on mental and physical health [77,78].

#### 4.1.4. Environment Dimension

The average scores for the environment dimension decreased slightly from 71.30 in 2022 to 68.09 in 2024 (Table 3), while increased variability indicated greater disparities. Research suggests this growing disparity may be attributed to socio-economic vulnerabilities and unequal access to quality resources in disadvantaged areas [79,80,81].

Table 4 highlights that rural respondents experienced the largest declines (71.54 ** to 67.57 *), reflecting challenges related to accessing quality resources. Rural populations are often the most affected by environmental stressors due to limited resources and reduced access to support infrastructure [82,83,84].

Young people aged 18–25 and women also reported notable declines. Marginalized groups, such as women and youth, are particularly vulnerable to environmental and social stressors, amplifying the negative effects on their well-being [85,86,87].

These trends align with studies that highlight the increased vulnerability of marginalized populations to environmental stressors. Chronic exposure to environmental stress can exacerbate inequalities and lead to long-term effects on the health and well-being of vulnerable populations [88,89,90].

#### 4.1.5. Subjective Perceptions

Interestingly, subjective responses regarding quality of life and satisfaction with health remained relatively stable across groups, with minor improvements for the 18–25 age group (3.29 ** to 3.41 *), while scores for other age groups remained constant. This stability can be explained by psychological resilience, defined as positive adaptation in the face of adversity, a phenomenon frequently observed in stressful contexts [91,92,93].

The discrepancy between objective declines and subjective stability suggests an adjustment of expectations. Studies show that people recalibrate their expectations and redefine success to adapt to adverse conditions, contributing to positive perceptions of quality of life despite objective challenges [94,95,96].

Psychological resilience is associated with various factors, including emotional flexibility and the use of effective emotion regulation strategies, which help individuals cope with adversity and maintain a positive perception of life [97,98,99].

### 4.2. Interpretation of Results and Implications for Public Health

The comparative study conducted in 2022 and 2024 highlights the significant impact of the Ukraine conflict on the quality of life (QoL) of Romanian citizens. This influence is reflected both in subjective perceptions and objective measurements, revealing a complex dynamic between the population’s perceptions and lived reality. The outbreak of an armed conflict near Romania’s borders has generated considerable psychological and social stress, amplified by anxiety related to potential escalations of the conflict [1,100].

Contrary to the objective declines observed, the stability of subjective perceptions in certain groups suggests the presence of resilience mechanisms that require more detailed exploration. Studies show that, in times of crisis, psychological resilience can act as a buffer against the negative effects of adversity, helping to maintain a more stable perception of quality of life [101,102].

The results show a pronounced sensitivity among young people aged 18 to 25, where the proportion of those perceiving the conflict as having a major impact on their lives increased from 39.5% in 2022 to 47.4% in 2024. Significant declines in the physical, psychological, and social dimensions suggest this group’s vulnerability to chronic stress and insecurity. Studies confirm that young people, constantly exposed to information about the conflict, may develop higher levels of anxiety and psychological stress, which affects their overall well-being [103,104].

The decline in the average score for the physical dimension, from 71.20 in 2022 to 63.74 in 2024, confirms that external pressures, such as geopolitical conflicts, have a tangible impact on physical health [1,105].

Female respondents reported an intensification in the perception of the conflict’s impact on their lives, and this is confirmed by significant declines in the psychological dimension, from 76.33 in 2022 to 69.85 in 2024. This decline reflects the cumulative effects of family and social responsibilities, as well as economic insecurity. Women are more susceptible to anxiety and stress due to these pressures, which increase their psychological vulnerability to adversity [101,106].

Residents in rural areas recorded the largest declines in the psychological and social dimensions. The proportion of those who believe the war affects their lives increased from 36.7% in 2022 to 43.5% in 2024. Determining factors include limited access to resources, social isolation, and economic pressures. Studies confirm that rural populations are often marginalized, which heightens their vulnerability in the context of geopolitical crises [1,107].

The discrepancy between the stability of subjective perceptions and the measured objective declines can be attributed to psychological resilience. This allows individuals to adjust expectations and find ways to cope with adversity. Access to information, social support, and resources is essential for developing this resilience [101,102].

The results confirm our research hypothesis, which states that at the time of this research (15 March–15 April 2024), there was a significant objective decline in the quality of life of young people in Romania compared to the onset of the conflict, although from a subjective perspective this decline is not perceived as significant due to the development of a resilience mechanism within the population.

These findings underline the need for tailored public health policies to specifically address the needs of the identified vulnerable groups, such as young people, women, rural populations, and those with middle and high school education. The vulnerabilities amplified by the Ukraine conflict indicate the necessity of clear interventions addressing both the objective dimensions of quality of life and subjective perceptions.

For young people, mental health programs represent a major priority. This category recorded the most significant declines in the psychological, social, and environmental dimensions, as shown by the data in the tables. A practical solution would be the development of accessible programs, such as free psychological counseling sessions in schools and universities or the creation of support helplines dedicated to young people affected by stress and anxiety. For example, in other European countries, programs like “Headspace” [108] in the UK have demonstrated the effectiveness of youth-oriented psychological support in preventing mental health problems. At the same time, to enhance social connections, it is essential to fund community initiatives that encourage interaction and civic participation, such as volunteer projects or cultural events [109,110,111].

Regarding women, who have experienced significant declines in the physical and psychological dimensions, interventions should include mental health and social support programs. For example, initiatives for support groups targeting women could provide a safe space to share experiences and access professional resources such as psychologists or social workers. In addition to mental health support, expanding access to preventive medical care and personalized treatments, particularly in rural areas, is necessary. Health education programs, such as campaigns on reproductive health, should be accessible and culturally tailored to reach women from diverse social backgrounds [112,113].

For the rural population, the data show that limited access to medical and social services has amplified declines in the quality-of-life dimensions. Investments in medical infrastructure are essential to reducing these disparities. For instance, building multifunctional medical centers in rural areas could ensure access to specialized consultations and basic treatments. Additionally, telemedicine programs could be an effective solution for residents in isolated communities, facilitating access to specialists through digital platforms. In Hungary, similar telemedicine projects have been successfully implemented, offering rapid access to diagnosis and treatment for rural populations. Social support could also be enhanced by creating community hubs to host educational, social, and economic activities [114,115,116].

For individuals with middle and high school education, whose level of awareness, knowledge, and perception of global realities is affected by military crises, such as the one in Ukraine, campaigns to promote security culture and awareness are necessary. The goal of such actions would be to increase citizens’ awareness of their role as security providers and to reduce the negative impact of military crises on their quality-of-life indicators [117,118].

Public health policies should address inequalities by focusing on equitable access to resources and support. It is essential for government strategies to include an integrated system for monitoring quality of life, tracking the evolution of both objective and subjective dimensions. This would allow for rapid adjustment of interventions based on the emerging needs of the population. A best practice example is the periodic implementation of national health and well-being surveys, such as the Eurobarometer, which provides valuable data for policy development.

In light of the above, the Ukraine conflict has amplified vulnerabilities among certain demographic groups, but the stability of subjective perceptions and selective improvements in some dimensions indicate significant potential for resilience. Through well-targeted public policies that combine infrastructure investments with psychological and social support programs, this potential can be strengthened to ensure better quality of life during periods of crisis and geopolitical instability.

Although previous results suggest a link between perceptions of quality of life and the conflict in Ukraine, we acknowledge that other contextual and individual factors may influence these perceptions, and our interpretations should be considered within the limits of the available data.

### 4.3. Limitations

This study has several limitations that must be acknowledged to contextualize its findings and interpret its conclusions with caution.

One significant limitation is the qualitative nature of the study and the lack of representativeness for the entire Romanian population. Although the online questionnaire involved a sufficient number of respondents aged 18 to 35, the sample does not fully reflect the socio-demographic diversity of Romanian society. This limitation reduces the generalizability of the study’s conclusions to a national level [119].

The method of distributing the questionnaire exclusively online introduces another major limitation. By restricting the participant pool to individuals with internet access, this approach potentially excludes disadvantaged or less connected groups, introducing selection bias. Moreover, the voluntary distribution of the questionnaire may result in subjective self-selection, where individuals with a specific interest in the subject are more likely to participate. This aspect could influence the overall validity of the conclusions and the diversity of perspectives represented in the study [120].

Another important constraint is the challenge of isolating the specific impact of the military conflict in Ukraine from other concurrent crises, such as the COVID-19 pandemic and the ongoing economic crisis. These overlapping events create interdependencies that shape respondents’ perceptions, complicating the analysis of the distinct influences of each crisis. For example, economic instability and public health concerns may amplify or obscure the effects attributed solely to the conflict. Online data collection methods, particularly those relying on platforms like Facebook, also introduce potential distortions due to selection bias and the digital context [121].

Additionally, a limitation lies in the study design, which did not allow for the inclusion of new questions in the second wave of data collection (2024). To ensure comparability between the responses from 2022 and 2024, the structure of the original research instrument was largely preserved. While this approach safeguards longitudinal consistency, it also restricts the ability to investigate additional variables that may have become relevant in the intervening period. For instance, questions exploring socio-economic, political, or psychological factors influencing both quality of life and perceptions of security could have provided more nuanced insights. This limitation underscores the difficulty in differentiating the specific effects of the Ukraine conflict from other contextual factors that have evolved over two years of socio-political change.

Lastly, while the study highlights potential associations between the Ukraine conflict and quality of life perceptions, it is essential to emphasize that these findings cannot be attributed solely to the conflict. Other contextual factors, such as media coverage, societal shifts, and personal experiences, may also contribute to the observed changes. Future studies could benefit from employing more flexible designs and comprehensive research instruments to disentangle these influences. Cross-country comparative analyses, as well as multi-method approaches, could provide a deeper understanding of the interplay between these factors and help generalize the findings beyond the specific Romanian context.

## 5. Conclusions

This research makes a significant contribution to understanding how modern conflicts affect civilian populations, particularly those geographically close to conflict zones. The study highlights the far-reaching consequences of such crises, which go beyond immediate concerns about physical safety to profoundly influence health, social cohesion, psychological well-being, and environmental quality [122,123]. By identifying how these dimensions interact, the research emphasizes the importance of understanding the specific vulnerabilities of various demographic groups, such as young people, women, rural populations, and those with lower levels of education, whose resilience or susceptibility is shaped by structural inequalities and access to resources.

The study provides an important foundation for the development of public health and security strategies, both nationally and internationally. The findings indicate that addressing the impact of regional conflicts on health and social cohesion requires more than reactive measures; they necessitate proactive policies that anticipate long-term needs and disparities. For instance, the psychological stress observed and the decline in quality of life among vulnerable groups underscore the need for targeted interventions in mental health and robust social support networks [124,125,126]. These efforts must align with broader security frameworks to ensure that public health and safety are treated as interdependent components of crisis response.

We also believe that targeted interventions addressing not only immediate needs but also building long-term resilience are imperative. Decision-makers have the responsibility to focus on equitable resource allocation, tailored public health strategies, and robust community development initiatives. By prioritizing inclusion and sustainability, these efforts can mitigate the cascading effects of modern conflicts and lay the foundation for a more resilient and cohesive society capable of facing future geopolitical and social challenges [127,128].

## Figures and Tables

**Table 1:** The socio-demographic data of the respondents.

Variables		18–25 Years		26–35 Years	
		2024	2022	2024	2022
Gender	Male	38.1	25.4	35.6	33.9
	Female	61.8	74.5	64.3	66
Environment	Rural	40.1	47.3	42.6	28.2
	Urban	59.8	52.7	57.3	71.3
Level of education	Middle and high school	84.7	60.9	26	21.9
	University	15.2	39	73.9	78

**Table 2:** Comparative analysis of perceptions regarding the conflict in Ukraine based on socio-demographic variables.

Socio-Demographic Data		Does the Conflict in Ukraine Influence Your Life in Any Way?					
		(1–2) [%]		(4–5) [%]		*t*-Test	
		2024	2022	2024	2022	t-Stat	*p*-Value
Age	18–25	19.8	16.4	47.4	39.5	−1.55	0.12
	26–35	17.0	21.5	41.7	38.8	−1.22	0.22
Gender	Male	16.9	15.8	53.0	47.6	1.96	0.05
	Female	19.4	19.3	39.8	35.9	−4.14	0.00
Environment	Urban	16.7	17.0	45.4	40.9	−0.53	0.59
	Rural	21.0	20.4	43.5	36.7	−2.12	0.03
Level of education	Middle and high school	17.6	17.1	45.2	39.2	−1.47	0.14
	University	18.5	19.4	43.9	39.3	−1.07	0.28

Chow Test: F-statistic = 0.612; degrees of freedom: df1 = 11 (number of predictors), df2 = 1572 (number of observations – 2 × k). Multiple regressions with interactions: 2022: Interaction between age and education level: coef. = 0.394 (*p* = 0.03, *p* = 0.03, *p* = 0.03). 2024: Interaction between age and education level: coef. = 0.432 (*p* = 0.03, *p* = 0.03, *p* = 0.03). Multiple regression analysis: 2022: Gender (female vs. male): coef. = −0.2811 (*p* = 0.001, *p* = 0.001, *p* = 0.001). 2024: Gender (female vs. male): coef. = −0.2362 (*p* = 0.005, *p* = 0.005, *p* = 0.005). Logistic regression: Used to estimate the effects of socio-demographic variables. Gender: coef. = −0.516 (*p* < 0.001, *p* < 0.001, *p* < 0.001). Year: coef. = 0.106 (*p* = 0.037, *p* = 0.037, *p* = 0.037).

**Table 3:** Descriptive statistics of health dimensions and quality of life between 2022 and 2024.

Descriptive Statistics				
	Minimum	Maximum	Mean	Std. Deviation
PHYS	7.14 (2024)/10.71 (2022)	100 (2024)/ 89.29 (2022)	66.76 (18.00) (2024)/61.64 (2022)	18.00 (2024)/13.49 (2022)
PSYCH	0.00 (2024)/16.67 (2022)	100	71.37 (2024)/73.16 (2022)	21.33 (2024)/15.05 (2022)
SOCIAL	0.00 (2024)/.00 (2022)	100	68.64 (2024)/74.62 (2022)	23.56 (2024)/23.07 (2022)
ENVIR	0.00 (2024)/3.13 (2022)	100	68.09 (2024)/71.30 (2022)	19.47 (2024)/18.60 (2022)

**Table 4:** Bivariate analysis of health dimensions and quality of life by age, gender, residence, and education level.

		Physical Health	Psychological Health	Social Relationship	Environmental Health	Quality of Life (QOL)	Health Satisfaction
Age	18–25	63.74 (17.09) * 71.20 (17.97) **	67.61 (21.38) * 76.54 (20.46) **	66.07 (21.38) * 73.55 (23.53) **	67.31 (19.01) * 72.32 (18.22) **	3.41 (1.17) * 3.29 (1.03) **	4.00 (1.04) * 4.11 (0.97) **
	26–35	69.91 (18.40) * 71.32 (18.61) **	75.29 (20.57) * 79.10 (19.81) **	71.32 (23.98) * 76.34 (22.23) **	68.90 (19.94) * 69.66 (19.11) **	3.34 (1.15) * 3.24 (1.07) **	3.93 (1.04) * 3.92 (0.99) **
Gender	Male	75.77 (17.97) * 75.20 (18.04) **	73.97 (20.94) * 80.45 (19.38) **	70.76 (23.67) * 75.50 (23.91) **	73.18 (18.71) * 73.49 (18.21) **	3.53 (1.20) * 3.47 (1.15) **	4.15 (1.00) * 4.15 (0.98) **
	Female	64.41 (17.62) * 69.66 (18.04) **	69.85 (21.42) * 76.33 (20.48) **	67.39 (23.43) * 74.26 (22.74) **	65.11 (19.31) * 70.43 (18.70) **	3.29 (1.13) * 3.19 (0.99) **	3.85 (1.05) * 4.00 (0.98) **
Envir,	Urban	67.34 (18.33) * 70.82 (18.74) **	71.73 (21.76) * 75.60 (20.50) **	69.31 (23.11) * 74.51 (22.65) **	68.46 (19.08) * 71.15 (18.27) **	3.40 (1.12) * 3.30 (1.02) **	3.95 (1.04) * 4.00 (1.00) **
	Rural	65.93 (17.52) * 71.89 (17.38) **	70.86 (20.72) * 80.37 (19.53) **	67.68 (24.19) * 74.77 (23.72) **	67.57 (20.03) * 71.54 (19.10) **	3.35 (1.22) * 3.23 (1.09) **	3.98 (1.05) * 4.09 (0.95) **
Level of education	Middle and high school	64.01 (17.04) * 71.76 (17.50) **	68.27 (21.10) * 77.50 (19.63) **	67.03 (22.25) * 74.20 (22.56) **	67.25 (18.17) * 72.52 (17.84) **	3.40 (1.12) * 3.28 (1.05) **	3.98 (1.02) * 4.11 (0.96) **
	University	59.80 (16.42) * 70.81 (18.79) **	70.45 (17.82) * 77.52 (20.78) **	70.68 (25.01) * 74.97 (23.52) **	69.16 (20.99) * 70.26 (19.18) **	3.35 (1.21) * 3.26 (1.05) **	3.95 (1.07) * 3.98 (0.99) **

* for 2024 and ** for 2022.

## Data Availability

Data can be requested from the corresponding author.
